# Intra-wound versus systemic vancomycin for preventing surgical site infection induced by methicillin-resistant *S. aureus* after spinal implant surgery in a rat model

**DOI:** 10.1186/s13018-023-03779-5

**Published:** 2023-04-13

**Authors:** Jian Wei, Hanwen Gu, Kai Tong

**Affiliations:** 1 Department of Orthopedic Surgery, Liuzhou People’s Hospital Affiliated to Guangxi Medical University, Liuzhou, 545006 China; 2 Department of Orthopedic Surgery, Zhongnan Hospital of Wuhan University, Wuhan, 430071 China

**Keywords:** Postoperative infection, Spinal implant surgery, Intra-wound vancomycin powder, Infection prophylaxis, Rat model

## Abstract

**Background:**

Systemic vancomycin administration pre-operatively for the infection prophylaxis of spinal implant surgery remains unsatisfactory. This study aimed to explore the efficacy and dosage of local use of vancomycin powder (VP) in preventing surgical site infections after spinal implant surgery in a rat model.

**Methods:**

Systemic vancomycin (SV; intraperitoneal injection, 88 mg/kg) or intraoperative intra-wound VP (VP0.5: 44 mg/kg, VP1.0: 88 mg/kg, VP2.0: 176 mg/kg) was applied after spinal implant surgery and methicillin-resistant *S. aureus* (MRSA; ATCC BAA-1026) inoculation in rats. General status, blood inflammatory biomarkers, microbiological and histopathological evaluation were performed during 2 weeks post-surgery.

**Results:**

No post-surgical deaths, wound complications and obvious signs of vancomycin adverse effects were observed. Bacterial counts, blood and tissue inflammation were reduced in the VP groups compared with the SV group. VP2.0 group showed better outcomes in weight gain and tissue inflammation than the VP0.5 and VP1.0 group. Microbial counts indicated that no bacteria survived in the VP2.0 group, whereas MRSA was detected in VP0.5 and VP1.0 groups.

**Conclusions:**

Intra-wound VP may be more effective than systemic administration in preventing infection caused by MRSA (ATCC BAA-1026) after spinal implant surgery in a rat model.

## Introduction

Surgical sites infection (SSI) is one of the most serious complications after spinal surgery, ranging from 0.3% to 20% [1, 2]. Staphylococcus aureus (*S. aureus*) and Staphylococcus epidermidis (*S. epidermidis*) are the major pathogenic bacteria [3], while approximately 23.1–75% of *Staphylococcus* clinically isolated are methicillin-resistant [3–5]. Studies indicated that systemic antibiotics administration such as cefazolin or vancomycin could not achieve a satisfactory effect of infection prophylaxis [6, 7]. Thus, intra-wound antibiotics powder such as VP in the surgical site for preventing SSI attracts the attention of the orthopedists.

Some clinical studies suggested that intra-wound VP intraoperatively in surgical sites of spinal surgery could significantly reduce the SSI rate without severe complications [8–15]. However, several studies reported that local use of VP in surgical wounds did not significantly alter the incidence of SSI in patients with surgically treated spinal pathologies [16–19]. All above studies illustrate the efficacy and safety of intra-wound VP in spinal surgery remain controversial. Moreover, no current guidelines are available for the use of intra-wound VP in preventing SSI, and no standard dosage for the drug exists. The dosage of intra-wound VP in most of the previous studies ranged from 0.5 g to 2 g, which was determined by the orthopedists, experience. The optimal dosage of intra-wound VP had never been evaluated in all the previous studies [10, 12, 20].

Herein, this study explored the dosage, efficacy and safety of intra-wound VP in preventing the post-surgical infection after spinal implant surgery in a rat model for purpose of providing evidence for clinical strategies.

## Materials and methods

### Animals and reagents

Wistar rats of SPF grade (male, aged 10 weeks, weighting 251 g ± 6 g) were obtained from the Center for Disease Control and Prevention (Hubei, China). The protocol of animal experiments was approved by the Committee on the Ethics of Animal Experiments of the School of Medicine, Wuhan University (No. AF339). All procedures of this study were designed and carried out following the Animal Research: Reporting of In Vivo Experiments (ARRIVE) and the Institutional Animal Care and Use Committee (IACUC) guidelines. All rats were housed in ventilated and sterilized cages at 22 ± 2 °C (humidity: 55 ± 5%) on a 12-h light/dark cycle with free access to standard chow and water and subjected to surgery after adaptive feeding for 1 week. Clinical-grade vancomycin hydrochloride for injection was obtained from Lilly (Japan).

### Bacteria

Individual colonies of MRSA (ATCC BAA-1026) were grown in tryptic soy broth (TSB; Solarbio, China), and the culture was diluted with PBS to a final bacterial load of 1.6 × 10^6^ CFU/100 µl, confirmed by viable plate count as we previously described [21].

### Study design

According to previously described rat spinal surgery models [22, 23], orthopedic-grade titanium alloy screws were chosen to simulate clinical spinal implant surgery. Sixty-five rats were randomly assigned to 5 groups: (1) CON (control, no antibiotics), *n* = 13. (2) SV: systemic vancomycin (88 mg/kg, intraperitoneal injection, half an hour pre-surgery, equivalent to 1 g in an adult human), (3) VP 0.5, VP 1.0 and VP 2.0: intra-wound vancomycin powder (44 mg/kg, 88 mg/kg and 176 mg/kg, respectively; once before the closure of incision intraoperatively). Doses of systemic vancomycin and intra-wound vancomycin were based on the dosage used in a prior rat model [24–26]. Table 1 reports the allocation of rats per group and the relative analysis.

**Table 1:** Allocation of animals per group and investigations

	Animals (*n* = 13 per group)												
Analyses	1	2	3	4	5	6	7	8	9	10	11	12	13
Spinal surgery + bacterial inoculation (Day 0)	x	x	x	x	x	x	x	x	x	x	x	x	x
Serum levels of vancomycin (Day 0)	x	x	x	x	x	x	x	x					
General status (Day 0, 4, 7, 14)	x	x	x	x	x	x	x	x	x	x	x	x	x
Incision examination (Day 14)	x	x	x	x	x	x	x	x	x	x	x	x	x
Tissue histology (Day 14)	x	x	x	x	x								
Implant SEM (Day 14)	x	x	x	x	x								
Microbiology (Day 14)						x	x	x	x	x	x	x	x
Liver and kidney histology (Day 14)	x	x	x	x	x	x	x	x	x	x	x	x	x
Serum ALT, AST, BUN and Cr (Day 14)	x	x	x	x	x	x	x	x	x	x	x	x	x

*ALT* alanine aminotransferase, *AST* aspartate aminotransferase, *Cr* creatinine. *UN* urea nitrogen. *SEM* scanning electron microscopy

### Surgical procedure

**Fig. 1:**
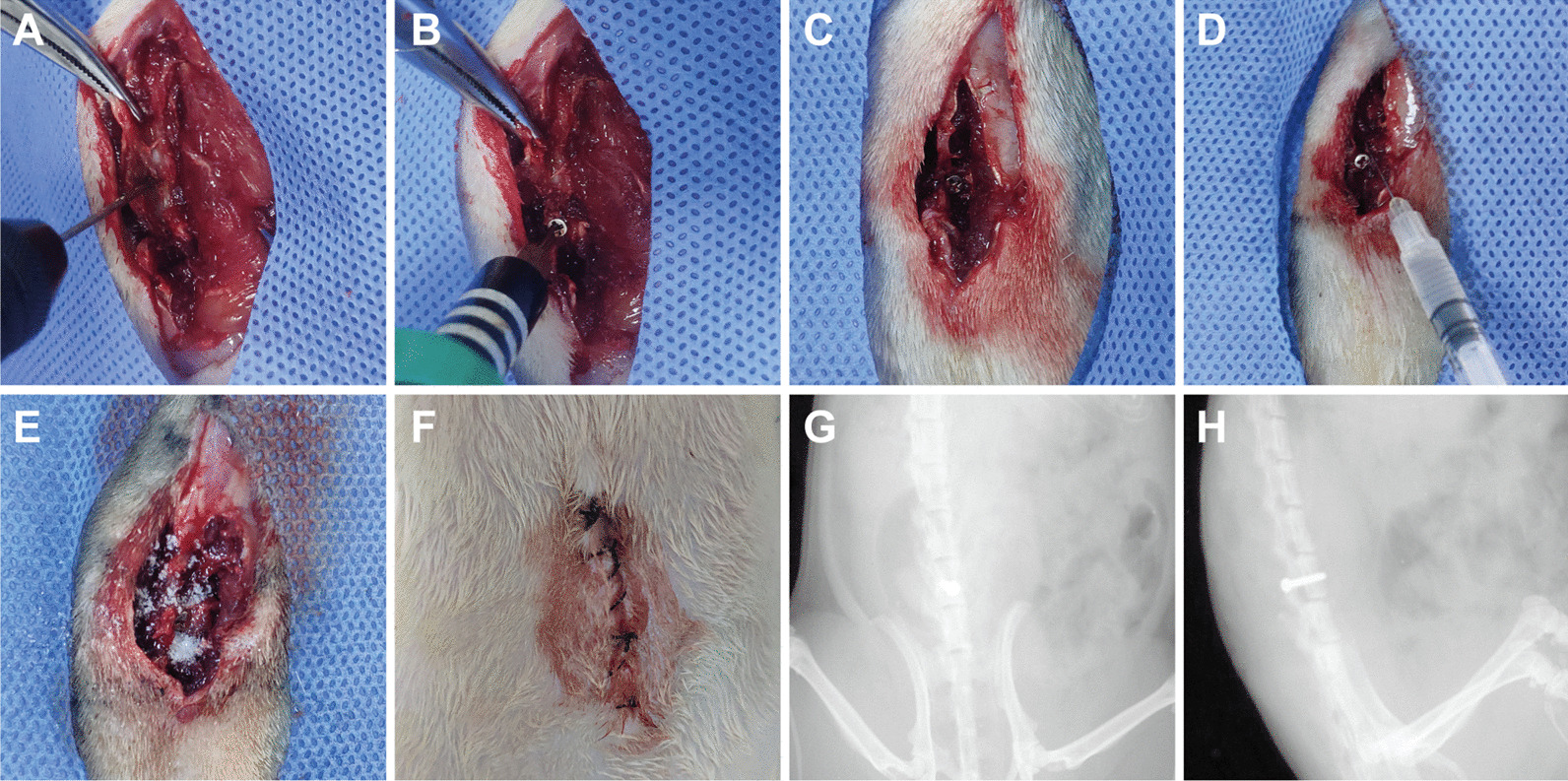
Surgical procedures for spinal implant surgery and modeling of the surgical site infection in a rat. **A**–**C** Spinal implant surgery procedures were performed in a rat model. **D** 1.6 × 10^6^ CFUs/100 µl of methicillin-resistant *S. aureus* (MRSA; ATCC BAA 1026) was inoculated in the surgical site. **E** Vancomycin powder was distributed in the wound during the surgery. **F**, **G** Anteroposterior and lateral radiographs of the implanted titanium alloy screws in the rat model were performed postoperatively (Bruker Xtreme BI, Germany; filter: 0.4 mm; 45 kVp; exposure time: 1.2 s; bin: 1 × 1 pixels; field of view [FOV]: 10 cm; f Stop: 2)

Spinal implant surgery was carried out on rats under general anesthesia using 2.5% isoflurane. A 3-cm midline longitudinal skin incision was made over the back at the level of the fourth and sixth lumbar vertebrae (L4-L6). An incision of the fascia and muscle was performed in order to expose the L4-L5 vertebra and spinous, with surrounding musculature and fascia separating. A 1.3-mm hole was drilled into the L5 vertebral pedicle, pointing obliquely to the L5 centrum, and an orthopedic-grade titanium alloy screw (width: 1.4 mm, length: 6 mm) was screwed into the hole (Fig. 1A–C); then, hemostasis was performed after saline irrigation. The surfaces of the screws and surrounding tissues were inoculated with 1.6 × 10^6^ CFUs of MRSA (ATCC BAA 1026) in 100 µl PBS (Solarbio, China; Fig. 1D). Intra-wound vancomycin powder was assigned to those rats of VP groups in the surgical sites (Fig. 1E). The surgical sites and overlying skin were closed with 4–0 sutures (Fig. 1F). X-ray was obtained following surgery to validate the correct location of the implants before continuing further experiments (Fig. 1G, H). Buprenorphine was used as postoperative analgesic (0.1 mg/kg/day) for 3 days. The rats were monitored daily for general status, incision healing, and vancomycin-related reaction any local soft tissue or systemic reaction related to the vancomycin and surgery. On post-surgical days 14, all rats were killed for blood collection and tissue harvest.

### General status and serum biomarkers

Body temperature and weight of rats in each group were measured preoperatively (day 0), and on post-surgical days 4, 7 and 14. Serum creatinine (Cr), urea nitrogen (UN), alanine aminotransferase (ALT) and aspartate aminotransferase (AST) were measured by ELISA kit (CUSABIO, China) on post-surgical days 14. The serum levels of vancomycin at 0.5 h, 2 h and 4 h after intra-wound VP application were detected by high-performance liquid chromatography-mass spectrometry (HPLC–MS, Thermo TSQ Quantis, USA).

### Scanning electron microscopy (SEM)

Samples preparations of the removal implants were referred to the previous protocols [27] and observed using Gatan digital camera system (Zeiss, Germany). The visual spherical structures of no surface deformities and approximately 1 µm in diameter were considered features of MRSA [28]. Five fields of view (FOV) on each implant were randomly observed under high magnification (× 5000) and counted.

### Incision healing and tissue histopathology evaluation

Incision healing was evaluated using a modified index score [29]. Gross tissue pathology was assessed on the base of the criteria of modified Rissing scale score [30], as follows: score 0, absence of abscess and ulcerative tissue; score 1, presence of minimal ulcerative tissue without abscess; score 2, tissue ulcerative and minimal abscess; score 3, abscess, sinus tract drainage or grossly purulent exudate; score 4, severe bone resorption, abscess. Soft tissue histopathology stained with hematoxylin and eosin (H&E) was performed to assess the tissue morphology of inflammation. Histological score of soft tissues referred to the modified Petty’s scale [31–33], as follows: score 0 (absent), absence of inflammatory cells; score 1 (mild), presence of occasional polymorph nucleated leukocytes; score 2 (moderate), scattered polymorph nucleated leukocytes and micro-abscesses; score 3 (severe), diffuse polymorph nucleated leukocytes with several micro- and great abscesses.

### Microbiological analysis

The centrum bone of L5 or surrounding soft tissues were harvested and homogenized by a tissue grinder (70HZ, 10 min; JXFSTPRP-48, China) with 5 ml of PBS on days 14, respectively. Each implant was placed in sterile PBS (2 ml), vortexed and sonicated to stimulate isolate of bacteria adherent to the implant [34]. Each tissue homogenate or sonicate solution was plated and CFUs counted after overnight culture at 37 °C. Bacterial colonies were identified as MRSA using Gram stain, catalase testing, plasma coagulase rapid agglutination tests and cefoxitin disc.

### Statistical analysis

Data were analyzed using SPSS software (versions 22.0, SPSS Inc., USA) and are presented as the means and standard errors of the means. Data were compared by analysis of variance (ANOVA) or unpaired 1-tailed Mann–Whitney test. *P* values of < 0.05 were considered significant.

## Results

### General status and serum inflammation marker

**Fig. 2:**
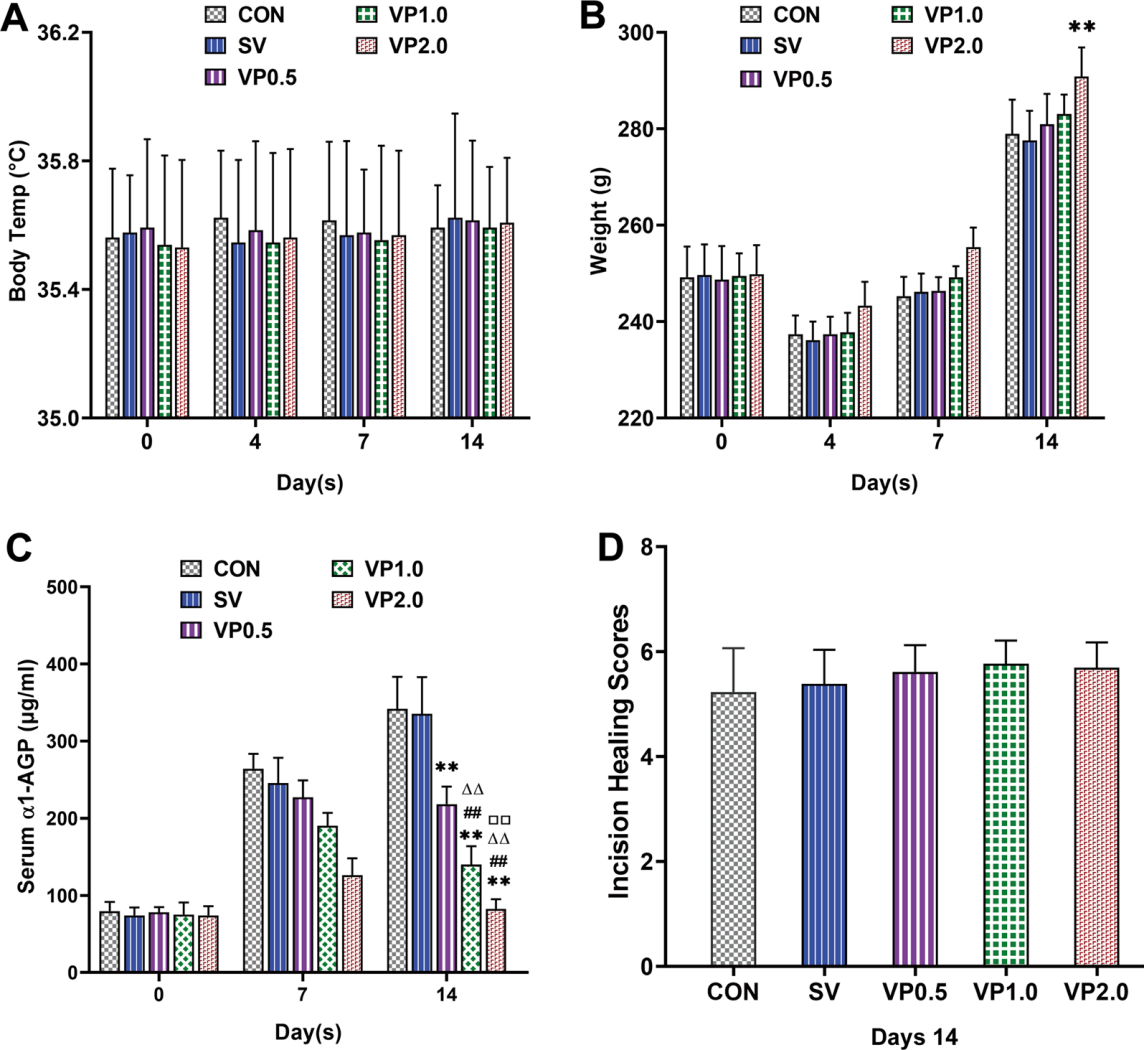
Changes in general status and serum inflammation marker throughout the experimental period. **A** Changes in body temperature during the study. Mean body temperature of rats in each group was measured preoperatively (day 0) and on post-surgical days 4, 7 and 14 using electronic thermometer. *n* = 13. **B** Mean body weight of rats in each group was measured preoperatively (day 0) and on post-surgical days 4, 7 and 14. *n* = 13. **C** The serum levels of α1-AGP during the study (pre-surgery [day 0] and on post-surgical days 7 and 14) in each treatment group. *n* = 8. **D** Incision healing scores of each treatment group on post-surgical days 14. *n* = 13. CON: control (no antibiotics); SV: system vancomycin (88 mg/kg, intraperitoneal injection, half an hour pre-surgery); VP 0.5: intra-wound vancomycin powder, 44 mg/kg, once before the closure of incision intraoperatively; VP 1.0: intra-wound vancomycin powder, 88 mg/kg; VP 2.0: intra-wound vancomycin powder, 176 mg/kg. Data were compared by analysis of variance (ANOVA) test. **P < 0.01 (compared with CON group), ^##^*P* < 0.01 (compared with SV group), ^ΔΔ^*P* < 0.01 (compared with VP0.5 group). ^□□^*P* < 0.01 (compared with VP1.0 group)

No statistical differences were detected in the body temperature among the 5 groups (Fig. 2A, P > 0.05). Bodyweight in the VP 2.0 group was greater than other four treatment groups (Fig. 2B, P < 0.05), whereas no statistical differences were observed in the CON, SV, VP 0.5 and VP 1.0 groups on postoperative days 14 (Fig. 2B, P > 0.05). Serum α1-AGP levels of rats in the CON and SV groups were significantly higher than in the VP group at 14 days postoperatively, and the VP 2.0 group was lower than that of the VP 0.5 and VP 1.0 groups (Fig. 2C, P < 0.01). The incisions of rats in each treatment group were healed on days 14 without wound ulceration or sinus tract, the incision healing scores were greater than 4, no statistical differences were observed between 5 groups (Fig. 2D, P > 0.05).

### Microbial counts

**Fig. 3:**
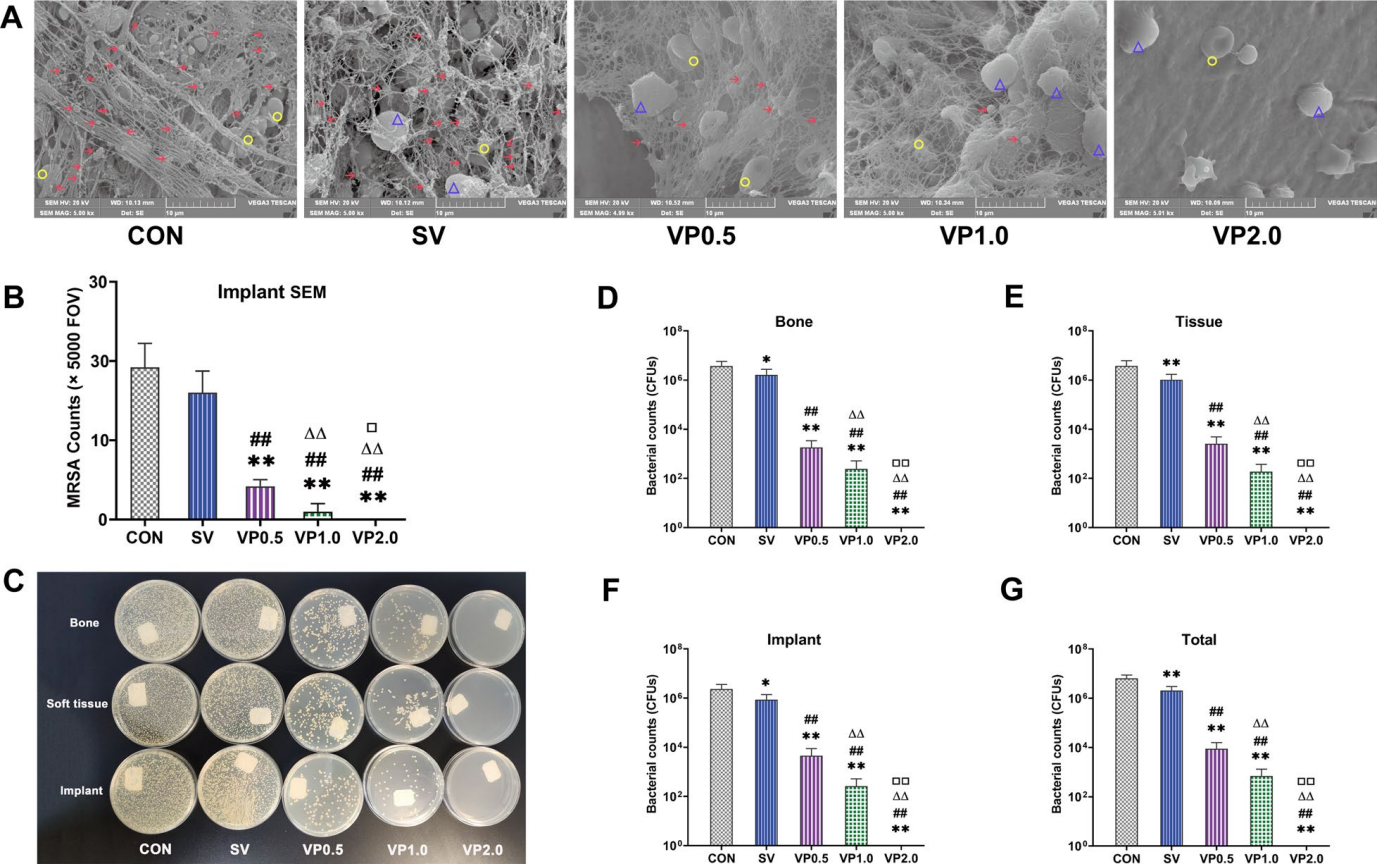
Microbiological evaluation in each treatment group. **A** SEM scanning of the implant with high magnification (× 5000). **B** Five fields of view (FOV) on each implant were randomly observed under high magnification (× 5000) and counted. *n* = 5. **C** Representative tryptic soy agar (TSA) plates of microbial culture of bone, soft tissue and implant in each treatment group. **D** The mean CFUs counts of the L5 centrum in each treatment group. **E** The mean CFUs counts of all soft tissues around the L5 centrum in each treatment group. **F** The mean CFUs counts of implant in each treatment group. **G** The mean CFUs counts of the whole animal in each treatment group. *n* = 8. CON: control (no antibiotics); SV: system vancomycin (88 mg/kg, intraperitoneal injection, half an hour pre-surgery); VP 0.5: intra-wound vancomycin powder, 44 mg/kg, once before the closure of incision intraoperatively; VP 1.0: intra-wound vancomycin powder, 88 mg/kg; VP 2.0: intra-wound vancomycin powder, 176 mg/kg. Data were compared by an unpaired 1-tailed Mann–Whitney test. *P < 0.05, **P < 0.01 (compared with CON group), ^##^*P* < 0.01 (compared with SV group), ^ΔΔ^*P* < 0.01 (compared with VP0.5 group). ^□□^*P* < 0.01 (compared with VP1.0 group). The red arrow indicates MRSA, the blue triangle indicates leukocyte, and the yellow circle indicates erythrocyte

A greater quantity of MRSA cells was observed on the implants in the CON and SV groups by SEM compared with the VP groups, surrounding with leukocytes or/and erythrocytes (Fig. 3A). No bacteria were observed in the VP 2.0 group, with fewer MRSA cells observed in the VP 1.0 group than that of VP 0.5 group (Fig. 3A, B). The representative tryptic soy agar (TSA) plates of bacterial culture are shown in Fig. 3C. The CFUs counts of each sample in the VP groups were significantly less than the CON and SV groups, and the CFUs of VP 0.5 and VP 1.0 groups were statistically higher than that of VP 2.0 group (Fig. 3D–G, P < 0.01).

### Tissue inflammation evaluation

**Fig. 4:**
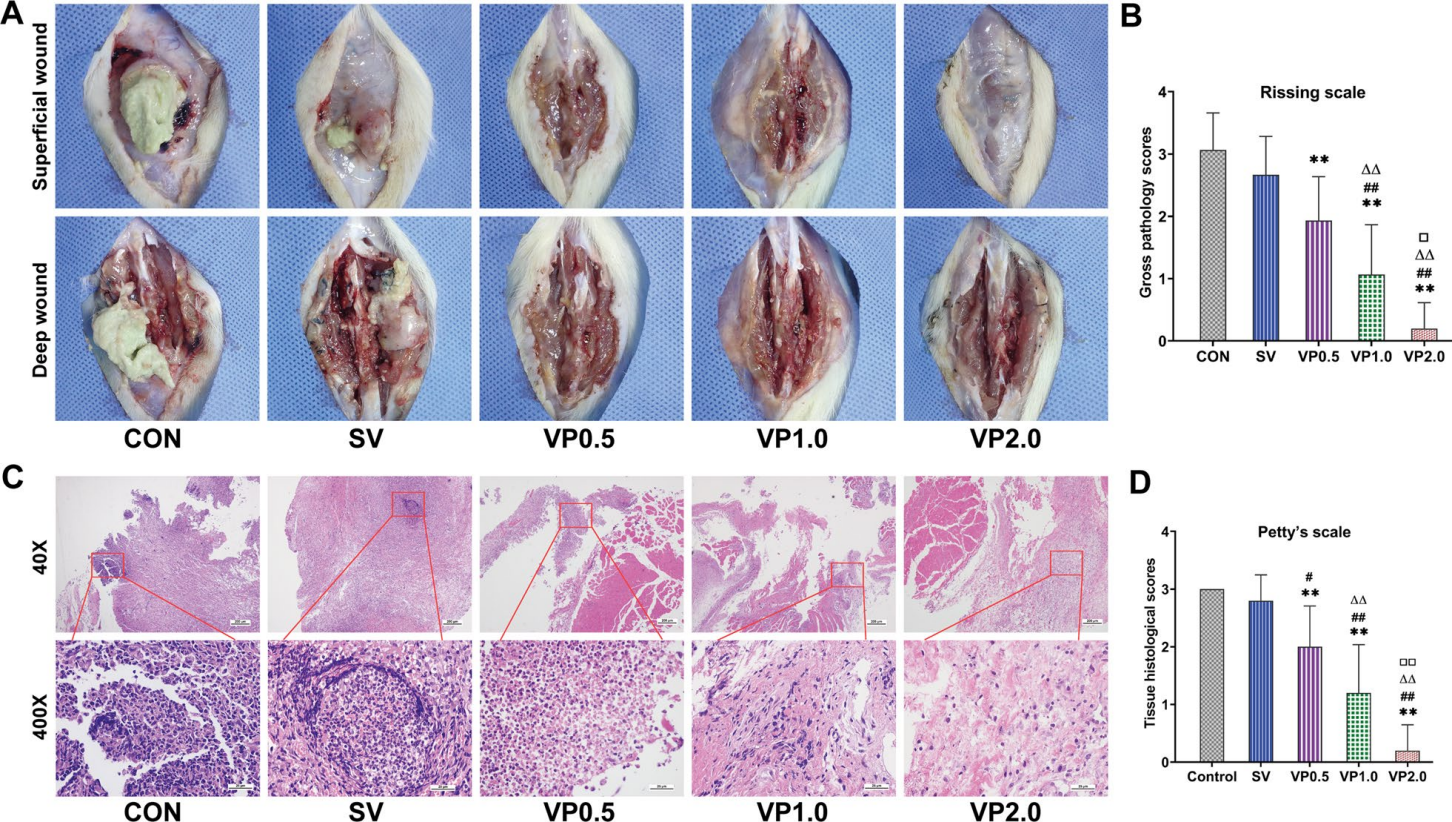
Gross pathology and histopathological assessment of the soft tissues in surgical sites. **A** Representative soft tissue of the lumbar spine appearance in surgical sites after post-surgical days 14 in each treatment group, after overlying skin was removed. **B** The gross tissue pathology scores based on the criteria of modified Rissing scale on post-surgical days 14 in each treatment group. *n* = 13. **C** Representative pathological H&E staining of the soft tissues in surgical sites on postoperative days 14 in each treatment group. **D** Mean soft tissue histological scores based on the criteria of modified Petty’s scale on postoperative days 14 in each treatment group. *n* = 5. CON: control (no antibiotics); SV: system vancomycin (88 mg/kg, intraperitoneal injection, half an hour pre-surgery); VP 0.5: intra-wound vancomycin powder, 44 mg/kg, once before the closure of incision intraoperatively; VP 1.0: intra-wound vancomycin powder, 88 mg/kg; VP 2.0: intra-wound vancomycin powder, 176 mg/kg. Data were compared by analysis of variance (ANOVA) test. **P < 0.01 (compared with CON group), ^#^*P* < 0.05, ^##^*P* < 0.01 (compared with SV group), ^ΔΔ^*P* < 0.01 (compared with VP0.5 group). ^□^*P* < 0.05, ^□□^*P* < 0.01 (compared with VP1.0 group)

Soft tissues ulceration and abscess formation were observed in the CON and SV groups by gross pathology of the surgical sites, whereas these changes were improved in the VP groups, especially in the VP 2.0 group (Fig. 4A). Modified Rissing scale scores in the VP 2.0 group were statistically lower compared with those of the CON, SV, VP 0.5 and VP 1.0 groups on post-surgical days 14 (*P* < 0.01, Fig. 4B). Acute purulent inflammation was observed in the surrounding soft tissues of CON and SV groups by histopathology, with abscess and infiltrations of immunoinflammatory cells (Fig. 4C). These changes were greatly reduced in the VP groups, among which the least inflammatory changes were observed in rats from the VP 2.0 group, with almost no inflammatory cells infiltration. Modified Petty’s scale scores in the VP 2.0 group were lower compared with those of the CON, SV, VP 0.5 and VP 1.0 groups (*P* < 0.01, Fig. 4D).

### Safety evaluation of intra-wound VP application in the spinal implant surgery

**Fig. 5:**
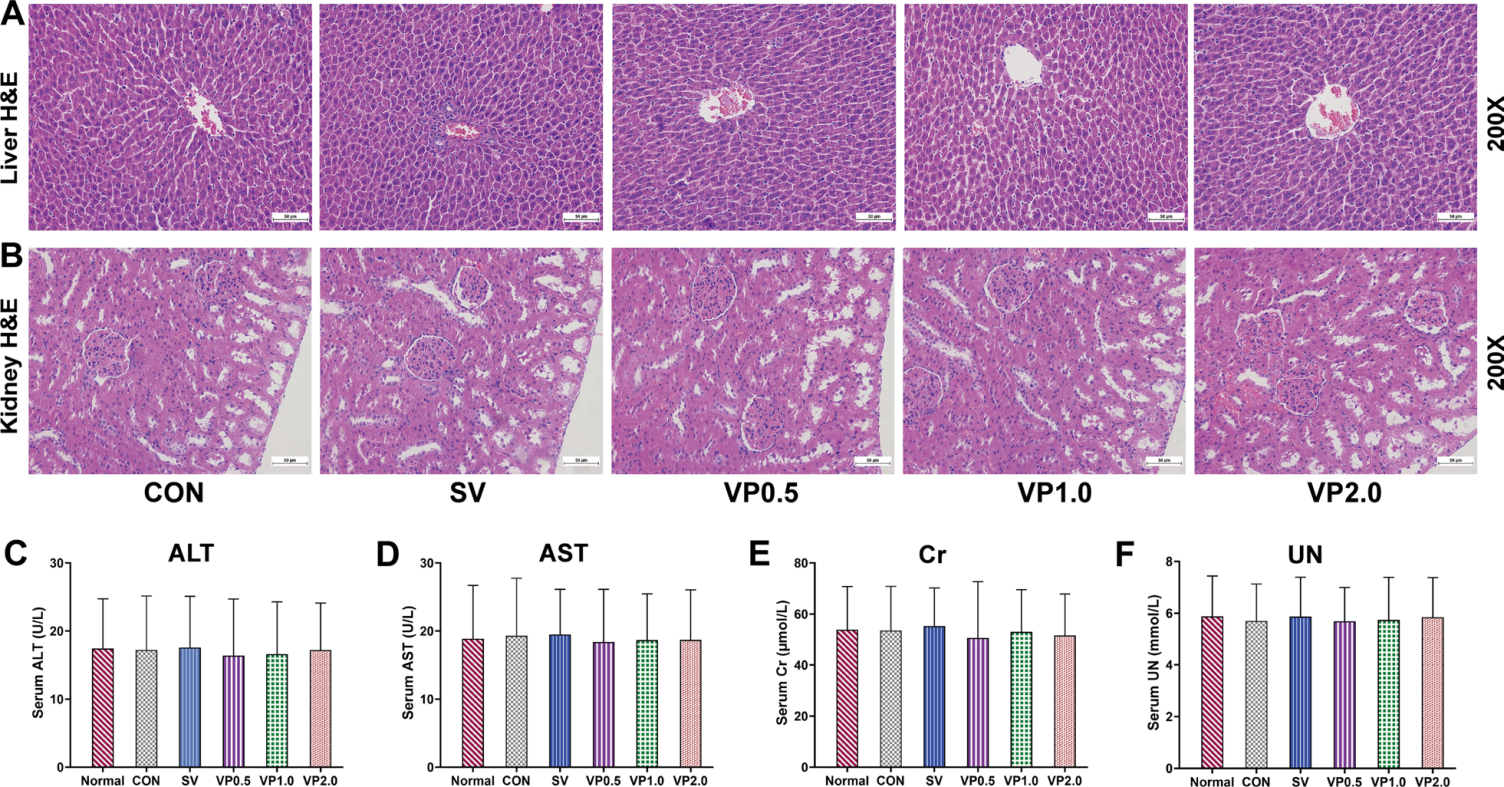
Safety evaluation of intra-wound VP in spinal implant surgery in each treatment group. **A** Representative pathological H&E staining of the kidney (× 200) on postoperative days 14 in each treatment group. **B** Representative pathological H&E staining of the liver (× 200) on postoperative days 14 in each treatment group. **C** Serum alanine aminotransferase (ALT) on postoperative days 14 in each treatment group. **D** Serum aspartate aminotransferase (AST) on postoperative days 14 in each treatment group. **E** Serum creatinine (Cr) on postoperative days 14 in each treatment group. **F** Serum urea nitrogen (UN) on postoperative days 14 in each treatment group. CON: control (no antibiotics); SV: system vancomycin (88 mg/kg, intraperitoneal injection, half an hour pre-surgery); VP 0.5: intra-wound vancomycin powder, 44 mg/kg, once before the closure of incision intraoperatively; VP 1.0: intra-wound vancomycin powder, 88 mg/kg; VP 2.0: intra-wound vancomycin powder, 176 mg/kg. Data were compared by analysis of variance (ANOVA) test. *n* = 13

No obvious structural changes were observed in the liver and kidney of each treatment group (Fig. 5A, B). No significant differences were observed among the CON, SV and VP groups in the serum Cr, UN, ALT and AST (Fig. 5C–F, P > 0.05). Serum vancomycin levels in the SV and VP groups were lower than the reported concentration necessary to induce nephrotoxicity (15–20 µg/ml) [35–39] (Table 2).

**Table 2:** Serum levels of vancomycin after spinal implant surgery (µg/ml)

Group	0.5 h	2 h	4 h
CON	0	0	0
SV	10.21 ± 1.09	3.55 ± 0.36	0.45 ± 0.06
VP 0.5	1.36 ± 0.30	0.55 ± 0.06	0.42 ± 0.08
VP 1.0	2.98 ± 0.35	0.74 ± 0.07	0.50 ± 0.06
VP 2.0	5.20 ± 0.66	1.39 ± 0.15	0.75 ± 0.09

*CON* control (no antibiotics); *SV* system vancomycin (88 mg/kg, intraperitoneal injection, half an hour pre-surgery); VP 0.5: intra-wound vancomycin powder, 44 mg/kg, once before the closure of incision intraoperatively; VP 1.0: intra-wound vancomycin powder, 88 mg/kg; VP 2.0: intra-wound vancomycin powder, 176 mg/kg. *n* = 8

## Discussion

Several clinical and basic studies indicated that the application of intra-wound VP in spinal implant surgery could reduce the incidence of post-surgical infection. Report from Lemans et al. refereed that the use of intra-wound VP was associated with a significant reduction in the post-surgical infection of spinal implant surgery [40]. Thompson et al. suggested that the application of VP in spinal surgery decreased the postoperative SSI rate (4.8% vs 13.8%; *P* = 0.038) compared with the untreated group [41]. Hida et al. studied 174 consecutive spinal surgery patients and found that intra-wound VP was effective in preventing SSI in cases with high risks of infection, without any side effects [42]. A meta-analysis of prospective and retrospective studies suggested that topical administration of VP could significantly decrease the incidence of post-surgical infection and was an effective and safe protocol to prevent infection after spinal operations [43].

However, the optimal and safe prophylactic dosage of intra-wound VP in the spinal implant surgery had never been evaluated in all these aforementioned studies. This rat-based study mimicked the use of intra-wound VP in the clinical spinal implant surgery and suggested that intra-wound VP resulted in less bacterial residue in surgical sites and milder inflammatory reaction in blood and tissues compared with systemic vancomycin. The dosage of 176 mg/kg of intra-wound VP (equal to 2.0 g in human) could eliminate the MRSA in the bone, soft tissue and implants of surgical sites. Therefore, the above study data indicated that intra-wound VP might replace systemic vancomycin as an effective protocols of infection prevention after spinal implant surgery.

Compared with systemic antibiotics, local use of antibiotics could reach higher concentrations (about 20 times of the minimal inhibitory concentration) while maintaining a safe systemic antibiotics concentration [44]. This local antibiotic administration achieves greater concentrations in critical areas such as postoperative seroma and ischemic tissue, while avoiding systemic toxicity reactions [45]. Due to the higher local antibiotic concentration, resistant bacteria may even be eliminated [46]. Given that surgical sites infections after spinal surgery caused by MRSA are particularly difficult to eliminate, local use of antibiotics, especially vancomycin, has been widely concerned and practiced by orthopedic scholars [47].

Some orthopedists have expressed concerning about the topical application of VP in spinal surgery. Horii et al. reviewed 2,859 spine surgical patients and found that intra-wound application of VP could not significantly decrease the incidence of surgical sites infections after spinal implant surgeries (1.73% *vs* 0.97%) compared with the untreated group [15]. Some studies suggested that intra-wound VP could not reduce the risk of deep SSI after spinal surgery, but might increase the propensity for gram-negative species [17, 48, 49]. A meta-analysis concerning that although intra-wound VP in spinal surgery decreased total infection rate (2.3% *vs* 3.8%; *P* < 0.05) compared with the untreated group, widespread use of intra-wound VP might increase the incidence of gram-negative and polymicrobial infection [50]. Martin found that no significant difference was observed in the incidence of peri-implanted infection rates with routine use of intra-wound VP [18]. Michael proposed that intra-wound VP could not completely eliminated SSI in high-risk patients; 16 patients (3.2%) in the cohort returned to the operating room for post-surgical infection [14].

No sufficient safety evaluation of intra-wound VP in the surgical sites of spinal operation was performed in these reports. In the current study, we found the serum vancomycin levels in the SV, VP 1.0 and VP 2.0 groups were higher than the MIC of ATCC BAA 1026 (2 µg/ml) within two hours after spinal implant surgery, but lower than that of inducing renal toxicity (15–20 µg/ml). In addition, no severe wound complications and obvious signs of vancomycin adverse effects in the structure and function of the liver and kidney were detected in rats that receiving intra-wound application of VP.

The current study does have limitations. First, the bacterial load of MRSA used in this study was higher than the clinical post-surgical infection cases, although the bacterial load was determined according to the prior rat studies that indicated a repeatable and stable postoperative spinal implant infection model [51, 52]. Second, 2 week of postoperative observation period may be too short for detecting chronic or delayed infection and osteogenic toxicity of vancomycin, further studies remain necessary.

In summary, in a rat model of a contaminated spinal implant surgery, use of intra-wound vancomycin powder could completely eliminate MRSA bacterial contamination at the dosage of 176 mg/kg (equal to 2.0 g in an adult). Animals treated with intra-wound vancomycin powder were more effective than systemic vancomycin in preventing MRSA contamination.

## Data Availability

All data generated or analyzed during this study are included in the main text. Additional data related to this article are available from the corresponding author upon reasonable request.
